# Effects of an Intravenous Infusion of Emulsified Fish Oil Rich in Long-Chained Omega-3 Fatty Acids on Plasma Total Fatty Acids Profile, Metabolic Conditions, and Performances of Postpartum Dairy Cows During the Early Lactation

**DOI:** 10.3389/fvets.2022.870901

**Published:** 2022-05-16

**Authors:** Matteo Mezzetti, Fiorenzo Piccioli-Cappelli, Andrea Minuti, Erminio Trevisi

**Affiliations:** ^1^Department of Animal Sciences, Food and Nutrition (DIANA), Facoltà di Scienze Agrarie, Alimentari e Ambientali, Università Cattolica del Sacro Cuore, Piacenza, Italy; ^2^Research Center Romeo and Enrica Invernizzi for Sustainable Dairy Production (CREI), Università Cattolica del Sacro Cuore, Piacenza, Italy

**Keywords:** essential fatty acids, inflammation, peripartum, immune modulation, anti-inflammatory, eicosapentaenoic acid, docosahexaenoic acid

## Abstract

A group of 10 multiparous Italian Holstein cows were housed in individual tied stalls and infused with 150 ml of saline (CTR; 5 cows), or of 10% solution rich in long-chained omega-3 fatty acids (n3FA; 5 cows) at 12, 24, and 48 h after calving. From −7 to 21 days from calving (DFC), the body condition score, body weight, dry matter intake (DMI), and milk yield were measured, blood samples were collected to assess the plasma fatty acids (FA) and metabolic profiles, and milk samples were collected to assess the milk composition. Data underwent a mixed model for repeated measurements, including the treatment and time and their interactions as fixed effects. Plasma FA profile from n3FA cows had lower myristic and higher myristoleic proportions, higher *cis*-11,14-eicosadienoic acid and monounsaturated FA proportions at 3 DFC, and lower *cis*-10-pentadecanoic proportion at 10 DFC. Besides these, n3FA cows had higher eicosapentaenoic (EPA) and docosahexaenoic (DHA) proportions (1.09 vs. 0.71 and 0.33 vs. 0.08 g/100 g), confirming the effectiveness of the infusion in elevating plasma availability of these FA. The plasma metabolic profile from n3FA cows revealed a tendency toward a lower concentration of reactive oxygen metabolites at 1 DFC and lower haptoglobin at 2 and 3 DFC, reflecting a mitigated inflammatory state. Furthermore, n3FA cows had a higher DMI during the first week of lactation. Higher DMI of n3FA could account for the changes detected on their plasma FAs, the higher milk yield they had at 1 and 2 DFC, the reduced lactose and urea nitrogen content in their milk. Higher DMI could also account for the lower plasma urea that n3FA cows had at 1 and 2 DFC, suggesting a lower amount of endogenous amino acids deserved to gluconeogenic fate. Milk from n3FA cows had lower rennet clotting time and higher curd firmness, which is probably driven by a higher EPA and DHA inclusion in the milk fat. Together, these outcomes suggest that the infusion exerts a short-term anti-inflammatory action on dairy cows at the onset of lactation.

## Introduction

Altered innate immune system functions exert a pivotal role in impairing the adaptation of dairy cows to new lactation (1–3). A dysfunctional immune system detrimentally affects performance and predisposes dairy cows to develop infectious and metabolic disorders (4–6). It has been hypothesized that the changes in the fatty acids (FAs) profile of plasma and cellular lipid fractions [i.e., decreased polyunsaturated FAs (PUFAs)/saturated FAs (SFAs) ratio] that are induced by the massive mobilization of fat depots at the onset of lactation, alter the leukocyte functions (7–10), and induce uncontrolled inflammatory responses in early lactating dairy cows (11–14). Among PUFAs, Omega-6 FAs (n6-FA) and Omega-3 FAs (n3**-**FA) are named “essential FAs” because they must be provided through the diet (15). Dairy cows commonly develop severe deficiencies for n3-FAs due to the massive inclusion of n6-FAs sources in the diets, leading to unbalanced PUFA ratios in all the body compartments, including immune cells phospholipid membranes (PLM) (16).

Supplementing n3-PUFAs to lower the plasma n6/n3 ratio represents a fascinating strategy to improve dairy cows' adaptation to the new lactation challenges through coping with the immune dysfunctions (14, 17–22). In fact, n3-FA are well known for their anti-inflammatory and immune-modulatory actions (i.e., shifted oxylipids profile in favor of anti-inflammatory mediators, reduced series 1 and 2 prostaglandins production, and improved leukocytes functions) (23–27). That said, supplementing the diet with feed sources containing n3-PUFAs is hindered by several limitations in dairy cows (i.e., feed intake depression, modifications in the profile of dietary FAs induced by rumen biohydrogenation, milk fat depression) (16, 28, 29), limiting dietary-administrable FAs to the rumen-protected forms. A promising strategy to cope with these limitations could be administering n3-PUFAs through venal infusion, but scarce are the studies performing a similar treatment on transition dairy cows. A major concern in supplementing huge amounts of fats by venal infusion during the early lactation could be aggravating the liver lipids load, sorting negative effects on animals' health (30, 31). Eicosapentaenoic acid (C20:5n-3 -cis–EPA) and docosahexaenoic acid (C22:6n-3 -cis – DHA) have the highest rate of inclusion in immune cell PLM among all the n3-PUFAs, representing the most valuable choice to effectively modulate immune cells functions through supplementing a small amount of lipids (15, 32, 33).

This experiment aimed to investigate the effect of infusing small amounts of EPA and DHA through the jugular vein of postpartum dairy cows on their performance and plasma profiles reflecting FA composition, inflammation, and metabolic conditions. The hypothesis was that venal infusion could alter the plasma FAs profile and that the anti-inflammatory properties of EPA and DHA could mitigate the inflammatory condition physiologically affecting dairy cows at the onset of lactation, positively affecting their performance.

## Materials and Methods

### Experimental Design and Animal Management

The trial was carried out at the Università Cattolica del Sacro Cuore research dairy barn (Experiment Station, San Bonico, Piacenza, Italy) following Italian laws on animal experimentation (DL n. 26, 04/03/2014) and ethics (Authorization of Italian Health Ministry N 1047/2015-PR). A group of 10 Italian Holstein dairy cows [number of lactations: 1.5 ± 0.7; milk yield in the last lactation: 11,547.8 ± 2,576 kg; average lactation length: 353.1 ± 54 days (mean ± SD)] were housed in individual tied stalls under a controlled environment (room temperature of 20°C, relative humidity of 65%, 14 h of light) from−35 to 21 days from calving (DFC). After calving, cows were milked two times a day at the stand at 4:00 am and 4:00 pm. All cows were individually fed a component diet of 2 equal meals of forages at 12-h intervals and 2–8 meals of concentrate supplied by a computer feeder. Before feed administration, residuals were individually weighed, and the amount of dry matter (DM) offered was changed based on the daily feed consumption. From−35 DFC to−7 DFC, animals received a hay-based ration with soybean meal and corn silage (Phase 1). Seven days before the expected day of calving, 2 kg of lactation concentrate was gradually added to the diet (Phase 2). After calving, the diet was enriched with 3 kg of alfalfa-dehydrated hay and the corn silage was increased to 20 kg/day by 2 kg/week. Grass hay was gradually reduced to 2–2.5 kg/day, and the concentrate was increased by 0.5 kg/day up to 14 DFC (Phases 3 and 4). From 15 DFC, the concentrate was increased gradually to meet the requirement of 1 kg per 3 kg of milk produced (Phase 5). The same batches of hay and corn silage were used during the trial. Feeds were collected every 2 weeks. After DM determination, samples were pooled for more analyses. Feed and diet composition are shown in Table 1.

**Table 1:** Composition and characteristics of the experimental diets fed to cows during the 5 experimental phases.

**Phase**	**Phase 1**	**Phase 2**	**Phase 3**	**Phase 4**	**Phase 5**
**Days from calving**	**-35-7**	**-7–0**	**1–7**	**8–14**	**15–21**
**Diet, % DM**					
**Item**					
Corn silage	23.8	23.9	33.2	29.9	28.0
Alfalfa hay	-	-	18.0	13.9	11.5
Grass hay	66.8	60.1	22.3	13.8	12.2
Concentrate (1–28 days from calving)	-	6.5	26.5	42.4	48.3
Concentrate (-35–0 days from calving)	9.4	9.5	-	-	-
**Concentrate composition, % DM**	**dry period**		**lactation**		
Corn flour	-			39.9	
Barley flour	-			1.4	
Soybean meal	90.5			13.1	
Sunflower meal	-			4.9	
Beet pulp	-			16.6	
Wheat bran	-			9.8	
Beet molasse slops	-			2.6	
Potato protein	-			2.2	
Hydrogenated palm oil	-			3.3	
Limestone	-			1.39	
Dicalcium phosphate	-			1.80	
Sodium bicarbonate	-			0.98	
Magnesium oxide	2.2			0.64	
Sodium chloride	1.4			0.32	
Supplement^a^	5.9			1.07	
**Chemical composition**					
NE_L_, Mcal kg of DM^−1^	1.31	1.36	1.54	1.64	1.67
Crude protein, % DM	11.4	12.3	15.3	16.3	16.7
Starch + sugar, % DM	13.4	15.0	21.5	24.0	24.9
Ether extract, % DM	1.71	2.01	2.83	3.50	3.77
NDF, % DM	49.4	47.3	38.1	35.1	34.0
MP^b^, % DM	9.4	10.1	12.4	13.6	14.0
RUP^b^, % DM	3.94	4.45	5.86	6.75	7.08

a *Supplements were composited to provide 150,000 UI of vitamin A, 10,000 IU of vitamin D, 200 mg of vitamin E, 100 mg of vitamin K, 100 mg of vitamin H1, 50 mg of vitamin B1, 0.5 mg of vitamin B12, 500 mg of vitamin PP, 4,000 mg of choline, 350 mg of Mn, 800 mg of Zn, 40 mg of Cu, 20 mg of I, 1 mg of Co, 1 mg Se*.

b *Estimated using NRC 2001*.

At−7 DFC, cows were enrolled in the experiment and divided into 2 homogeneous groups by body condition score (BCS), the number of lactations, milk yield, and age. Both groups received a jugular infusion at 12, 24, and 48 h after calving. The control group (CTR; 5 cows) was infused with 150 ml of sterile saline solution (Liofilchem, Roseto degli Abruzzi, Italy), while the group treated with omega-3 FA (n3-FA; 5 cows) was infused with 150 ml of a lipid supplement for parenteral nutrition (Omegaven, Fresenius Kabi, Italy). According to the manufacturer's instruction, 100 ml of the lipid supplement provided 112 kcal and consisted of 10 g of highly purified fish oil (containing 1.25-2.82 g of EPA, 1.44-3.09 g of DHA), 0.015-0.0296 g of α-tocopherol, 2.5 g of glycerol, 1.2 g of egg purified phosphatide, sodium oleate, sodium hydroxide, and physiological solution.

**Figure 1:**
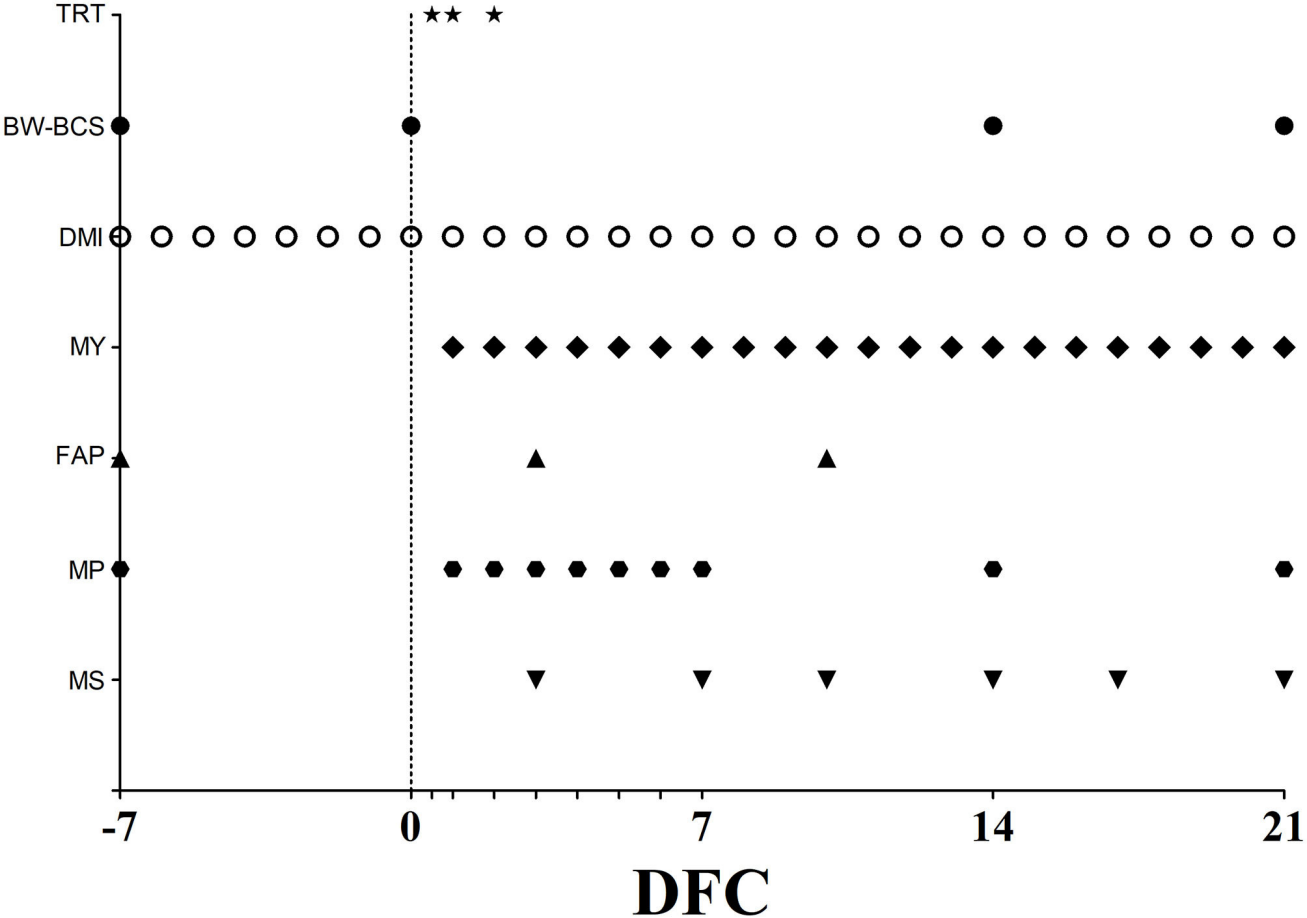
Scheduled time points, expressed as day from calving (DFC), for jugular infusion of physiological solution or emulsified fish oil (TRT), body weight and body condition score determination (BW–BCS), dry matter intake measurement (DMI), milk yield recording (MY), blood samples collection for plasma fatty acids profile (FAP) and plasma metabolic profile assessment (MP), and milk sample collection (MS). Empty ticks indicate 0.5, 1, 2, 3, 4, 5, and 6 DFC, respectively.

Between−7 and 21 DFC, periodic checks were performed, and blood samples were regularly collected according to the schedule shown in Figure 1 and described in the following sections.

### Health Status

The health conditions of cows were monitored daily from−35 to 21 DFC. Rectal temperature was measured daily with a thermometer. Mastitis was diagnosed by visual evaluation of abnormal milk from each quarter and somatic cell count (SCC) analysis in suspicious cases. The retained placenta was diagnosed when the fetal membranes were not expelled within 24 h after calving. Endometritis and metritis were diagnosed according to Sheldon et al. (34). Diarrhea was diagnosed by a visual evaluation of fecal consistency and color according to the fecal score method (35), assuming diarrheic feces to have a fecal score ≤2.

### Bodyweight, Body Condition Score, Dry Matter Intake, and Milk Yield

At−7, 0, 14, and 21 DFC, the body weight was measured in the morning, before feeding administration, with a single walk-in scale, and the same operator determined the BCS with a 1–4 scale (36). BCS variation (**Δ**BCS) was calculated as the difference between the value registered on calving day and those registered at 21 DFC. The individual daily intake was measured by weighing the amount of feed administered and its residual. The daily dry matter intake (DMI) was calculated based on the dry matter content of each feed and used to create an average weekly value. Milk yield was weighed after each milking.

### Blood Sample Collection

Blood samples were harvested through jugular venipuncture in evacuated heparinized tubes (BD Vacutainer; BD and Co., Franklin Lakes, NJ, United States) before the morning feeding. Samples were processed according to Calamari et al. (37). After processing, plasma samples were stored at−20°C. After all blood samples were collected, all frozen samples were thawed and used for different assays, as shown in Figure 1 and described in the following section (maximum storage time of 6 months).

#### Plasma Fatty Acids Profile

Samples were collected at−7, 3, and 10 DFC. Extraction and methylation of plasma FAs were performed following Shingfield et al. (38). Briefly, total lipids were extracted from plasma with chloroform/methanol (2:1, vol/vol). Fatty acids were methylated by *in situ* trans-esterification with 0.5 N methanolic NaOH and 14% boron trifluoride in methanol. After adding a saturated solution of NaCl, methyl-ester of FAs was extracted with 1 ml of hexane, and a fraction was transferred to gas-chromatography vials. FAs were separated using a gas chromatographer (Agilent 7250 A, Palo Alto, CA) fitted with a CP-Sil88 column (100 m x 0.25 mm ID, 0.20 μm film thickness Agilent Technologies). Helium was used as carrier gas (flow 1 ml/min). A 2 μl of the sample was injected with a split ratio of 1:25. The oven's initial temperature was 50°C, held constant for 1 min before being increased to 175 by 25°C/min. The temperature was then increased by 4°C/min until 230°C and held constant for 5 min. Peaks of individual FAs were identified and quantified using a 37 FAME standard (Supelco, Bellefonte, PA, United States). Rumenic acid and other conjugated linoleic acid (CLA) isoforms were individuated utilizing the combination of relative standards (Sigma–Aldrich). Individual FAs were expressed as their molar proportion on the total circulating FAs and grouped into 4 functional classes based on the number of double bonds. Furthermore, the total amount of SFAs, unsaturated FAs, MUFAs, PUFAs, SFAs/UFAs, and n6/n3 ratios were calculated.

#### Plasma Metabolic Profile

Samples were collected at−7, 1, 2, 3, 4, 5, 6, 7, 14, and 21 DFC. A clinical auto-analyzer (ILAB-650, Instrumentation Laboratory, Lexington MA, United States) was used to determine the concentration of glucose, non-esterified fatty acids (NEFA), beta-hydroxybutyrate (BHB), urea, creatinine, Zn, aspartate amino transferase-glutamate oxaloacetate transaminase (AST-GOT), gamma-glutamyl transferase (GGT), alkaline phosphatase (ALP), total protein, haptoglobin, ceruloplasmin, albumin, total bilirubin, cholesterol, and globulin following Calamari et al. (37). Furthermore, reactive oxygen metabolites (ROM) were determined according to Jacometo et al. (39), paraoxonase (PON) according to Bionaz et al. (40), thiol groups (SHp) according to Minuti et al. (41). Calibration was performed according to commercial standards for Zn, ceruloplasmin, albumin, protein, bilirubin, ALP, NEFA, BHB, and ROMt. Calibration for the remaining indicators was performed through internal standards. Four different quality controls were used to test the repeatability and precision of each parameter during each assay. Retinol, tocopherol, and beta-carotene were analyzed by reverse-phase HPLC (LC-4000, Jasco Europe, Carpi MO, Italy), as described by Jahan et al. (42). Further details on the analytical procedures adopted in blood analysis are reported in Supplementary Table 1.

### Milk Sample Collection and Analysis

At 3, 7, 10, 14, 17, and 21 DFC, milk samples were collected into 100-ml polypropylene bottles (International Scientific Supplies Ltd., Bradford, United Kingdom) during the morning milking (Figure 1). Butterfat, protein, lactose, casein content, and titratable acidity were measured by using infrared instrumentation (MilkoScan FT 120, Foss Electric, Hillerød, Denmark) according to Calamari et al. (43) and Chessa et al. (44). Rennet coagulation properties (Rennet clotting time–rCT–and curd firmness at 30 min–a30) were also measured using a computerized renneting meter (Foss Electric), where 12 ml of milk was heated to 35°C, and 240 μl of Rennet (Hansen standard with 63% chymosin and 37% pepsin, Pacovis Amrein AG, Bern, Switzerland) diluted to 1.6% (wt/wt) in distilled water was added to milk. Urea nitrogen in skimmed milk was determined by a spectrophotometric assay using a urea nitrogen kit (cat# 0018255440, Instrumentation laboratory, Milano, Italy) along with a clinical auto-analyzer (ILAB-650, Instrumentation Laboratory, Lexington MA, United States). The true protein value was calculated as the difference between the protein and urea nitrogen content, the output of fat and protein, and the fat to protein ratio were also calculated. The energy corrected milk (ECM) was calculated according to Sjaunja et al. (45), and the gross feed efficiency was calculated as the ratio between ECM and DMI. The SCC was determined using an optofluorometric method with an automated cell counter (Fossomatic 180, Foss Analytics, Hillerød, Denmark) and expressed as a linear score following Wiggans and Shook (46).

### Statistical Analysis

The data were analyzed in SAS software version 9.4 (SAS Inst. Inc., Cary, NC, United States) and presented in the graphs and tables as means and pooled standard error for individual means of treatment over time. Before analysis, the normality of data distribution was verified for each parameter by evaluating skewness and kurtosis according to the Shapiro test in SAS.

The prevalence of health problems recorded during the study was evaluated by χ2 analysis (Freq procedure, SAS Institute, Inc., Cary, NC, United States).

The data on DMI, body weight, BCS, milk yield, plasma FAs and metabolic profiles, and milk quality measurements underwent ANOVA, using a mixed model for repeated measures (Glimmix procedure, SAS Inst. Inc.) following Littell et al. (47). The statistical model included the fixed effect of treatment (TRT; CTR and n3FA), time, and their interaction (TRT X time). For DMI, the time effect was considered the average weekly value; for other parameters (milk yield, body weight, BCS, plasma FAs and metabolic profiles, and milk quality measurements), it was considered a single DFC. The time was considered a random effect within the cow, and the cow was assumed as a residual subject. The analysis was carried out using two covariance structures with their heterogeneous counterparts: autoregressive order and compound symmetry. These were ranked according to their Akaike information criterion, with the structure having the lowest Akaike information criterion being chosen (47). A preliminary analysis was conducted, and all parameters were covariate using data collected at−7 DFC as the baseline. The covariate was included in the final model only for parameters with a significant covariate effect in the preliminary analysis, adopting *P* ≤ 0.1 as a cutoff for covariate inclusion.

After the analysis, the residuals were plotted to assess for model assumptions of normality and homoscedasticity. The *post-hoc* comparison between treatments was done using the F-test and discussed when the main effect's *P*-value was ≤ 0.05. The main effects at *P* ≤ 0.10 are discussed in the context of tendencies. Differences between treatments at single time points are discussed at *P* ≤ 0.10 for the main interaction effect.

## Results

Excluding the FA plasma profile, the time effect was highly significant (*P* < 0.01) for most of the other parameters in the study. Thus, the time effect will be presented only in the Results section for the plasma FA profile.

### Health Status, Body Weight, Body Condition Score, Dry Matter Intake, and Milk Yield

**Figure 2:**
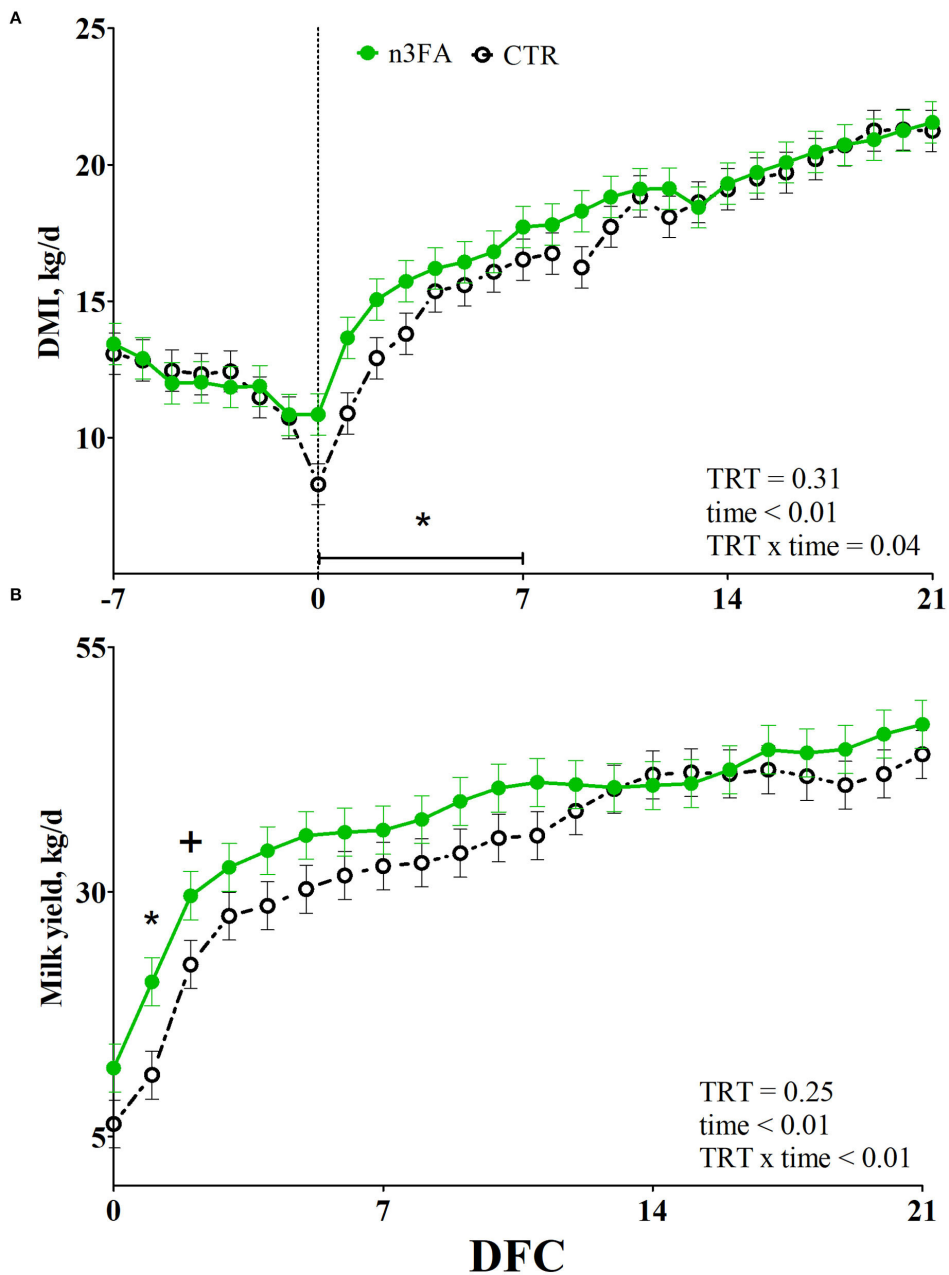
Average week values of dry matter intake (**DMI**; **A**) and daily values of milk yield **(B)** in dairy cows infused with 150 ml of sterile saline (CTR; black dotted line) or 150 ml of a 10% solution containing purified fish oil rich in long-chained omega-3 fatty acids (n3FA; solid green line) at 12, 24, and 48 h after calving. TRT is treatment effect; TRT × time is treatment × time interaction effect (* is *P* < 0.05; + is *P* < 0.10).

None of the cows had any diseases before parturition, and no difference between groups appeared for the incidence of diseases after calving (Table 2). While body weight was higher in n3FA than CTR group (*P* = 0.04), there was no difference between the groups that appeared for BCS or ΔBCS (Table 3). DMI and milk yield were affected by a TRT × time interaction (*P* = 0.04 and *P* < 0.01; Figures 2A,B). Compared to CTR, the n3 FA group had a higher DMI during the first week after calving (*P* < 0.05), a higher milk yield at 1 DFC (*P* < 0.05), and a tendency toward a higher milk yield at 2 DFC (*P* < 0.1).

**Table 2:** Incidence of diseases observed between −35 and 21 days from calving in dairy cows infused with 150 ml of sterile saline or 150 ml of a 10% solution containing purified fish oil rich in long-chained omega-3 fatty acids at 12, 24, and 48 h after calving.

**TRT^**a**^**	**Milk fever**	**Diarrhea**	**Dermatitis**	**Healthy**	**Sick**
CTR	0	1	1	4	1
n3FA	0	0	1	4	1
χ^2^	1.0	0.50	0.55	1.0	1.0

a *Treatment (CTR is cows receiving physiological solution; n3FA is cows receiving emulsified fish oil as intrajugular infusion)*.

**Table 3:** Trends of body weight and body condition score in dairy cows infused with 150 ml of sterile saline or 150 ml of a 10% solution containing purified fish oil rich in long-chained Omega-3 fatty acids at 12, 24, and 48 h after calving.

**Item, Unit^**1**^**	**TRT^**2**^**	**Days from calving**				**SEM^**3**^**	***P*** **-Value**		
		**-7**	**0**	**14**	**21**		**TRT^**2**^**	**Time**	**TRT X time^**4**^**
BW^c^, kg	CTR	713.0	643.4	612.4	586.6	10.2	0.04	<0.01	0.64
	n3FA	725.2	667.8	643.4	622.8	10.2			
BCS^c^, -	CTR	2.51	2.45	2.34	2.20	0.04	0.87	<0.01	0.56
	n3FA	2.47	2.46	2.39	2.21	0.04			
ΔBCS, -	CTR	-	-	-	0.25	0.06	0.91	-	-
	n3FA	-	-	-	0.24	0.06			

1 *BW, body weight; BCS, body condition score; ΔBCS, Difference between BCS value at calving and 21 DFC*;

c *parameter was covariate on−7 DFC value*.

2 *Treatment (CTR is cows receiving physiological solution; n3FA is cows receiving emulsified fish oil as intrajugular infusion)*.

3 *Standard error = largest standard error for the fixed effects*.

4 *Treatment × time interaction effect*.

### Plasma Fatty Acids Profile

#### Saturated Fatty Acids

**Figure 3:**
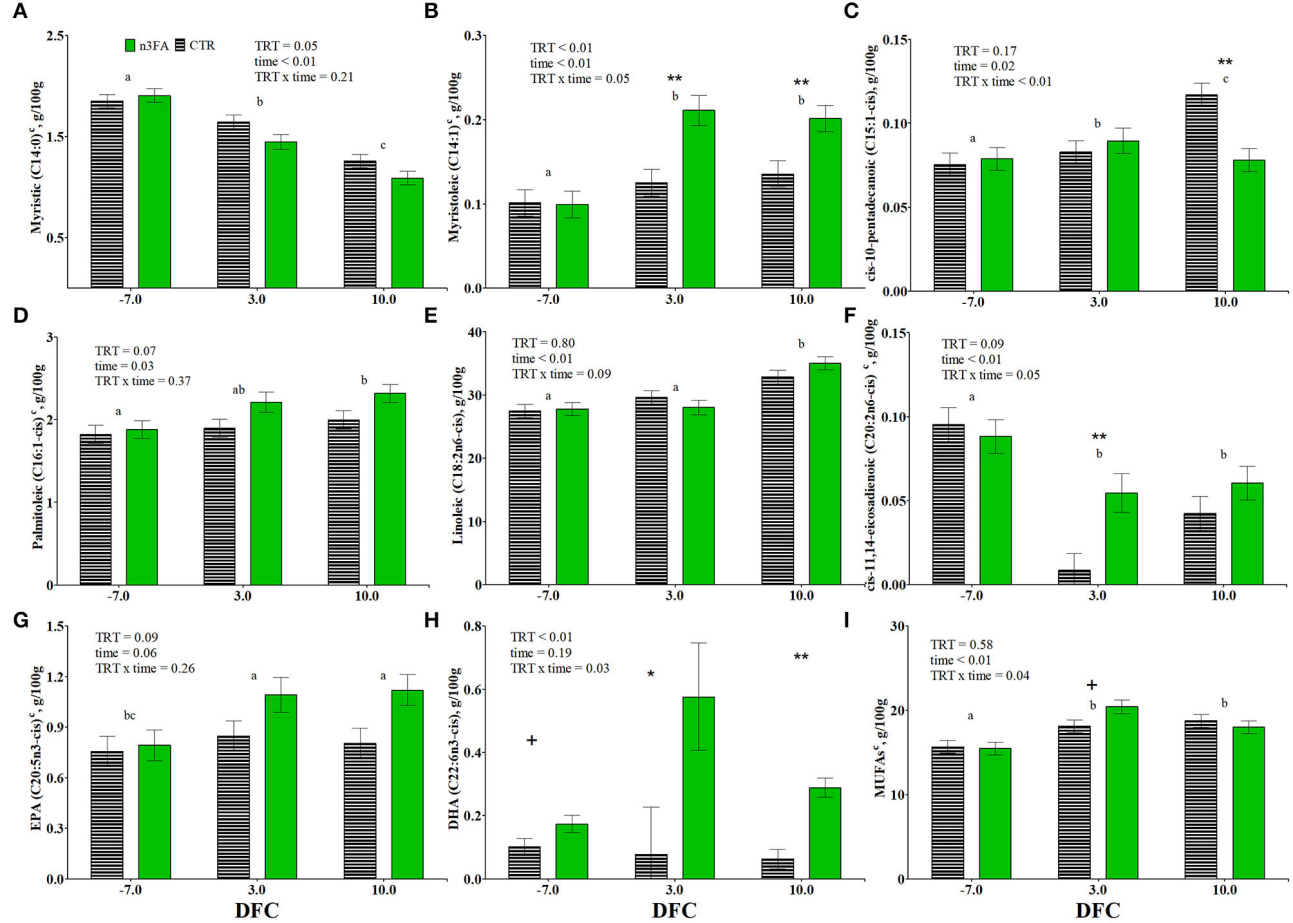
Time course of plasma relative abundances of myristic acid (C14:0; **A**), myristoleic acid (C14:1; **B**), cis-10-pentadecanoic acid (C15:1-cis; **C**), palmitoleic acid (C16:1-cis; **D**), linoleic acid (C18:2n6-cis; **E**), cis-11,14-eicosadienoic acid (C20:2n6-cis; **F**), eicosapentaenoic acid (C20:5n3-cis; **G**), docosahexaenoic acid (C22:6n3-cis; **H**), and total monounsaturated fatty acids (MUFAs; **I**) in dairy cows infused with 150 ml of sterile saline (CTR; black dotted line) or 150 ml of a 10% solution containing purified fish oil rich in long-chained omega-3 fatty acids (n3FA; solid green line) at 12, 24, and 48 h after calving. TRT is treatment effect; time is time effect (a, b, c signify differences between time points at *P* < 0.05. Time points having the same letters are equal), TRT × time is treatment × time interaction effect (** is *P* < 0.1; * is *P* < 0.05; + is *P* < 0.10), c parameter was covariate on−3 DFC value.

While the proportion of butyric (C4:0) and capric acid (C10:0) decreased at 10 DFC, those of stearic (C18:0), arachidic (C20:0), behenic (C22:0), and tricosanoic (C23:0) acids decreased at 3 DFC and remained constant during lactation. Lauric (C12:0), myristic (C14:0), pentadecanoic (C15:0), and heptadecanoic (C17:0) acids decreased throughout the experimental period (Table 4, Figure 3A). The proportion of myristic acid was lower in the n3FA than in the CTR group (*P* = 0.05; Figure 3A). But no differences over time or between groups appeared for the concentration of caproic acid (C6:0), caprylic acid (C8:0), undecanoic acid (C11:0), palmitic acid (C16:0), nonadecylic acid (C19:0), and lignoceric acid (C24:0) (data not shown).

**Table 4:** Trends of plasma fatty acids profile in dairy cows infused with 150 ml of sterile saline or 150 ml of a 10% solution containing purified fish oil rich in long-chained omega-3 fatty acids at 12, 24, and 48 h after calving.

**Item, Unit^**1**^**	**TRT^**2**^**	**Days from calving**			**SEM^**5**^**	***P*** **-Value**		
		**-7**	**3**	**10**		**TRT^**2**^**	**Time^**3**^**	**TRT X Time^**4**^**
**Saturated fatty acids, g/100 g**								
Butyric	CTR	3.20	4.43	3.02	0.55	0.24	0.07	0.44
	N3FA	2.98	3.05	2.26	0.60			
		ab	a	b				
Capric^c^	CTR	1.04	1.15	0.77	0.08	0.56	<0.01	0.93
	N3FA	1.03	1.08	0.73	0.09			
		a	a	b				
Lauric^c^	CTR	2.23	1.95	1.49	0.37	0.96	0.01	0.32
	N3FA	2.68	1.64	1.38	0.37			
		a	b	c				
Pentadecanoic^c^	CTR	1.08	0.68	0.58	0.04	0.62	<0.01	0.60
	N3FA	1.07	0.73	0.62	0.04			
		a	b	c				
Heptadecanoic^c^	CTR	0.72	0.53	0.50	0.03	0.29	<0.01	0.10
	N3FA	0.68	0.63	0.53	0.04			
		a	b	c				
Stearic^c^	CTR	16.3	12.6	13.5	0.79	0.35	<0.01	0.41
	N3FA	15.6	13.2	11.7	0.91			
		a	b	b				
Arachidic^c^	CTR	0.15	0.05	0.05	0.01	0.19	<0.01	0.16
	N3FA	0.13	0.09	0.07	0.02			
		a	b	b				
Behenic^c^	CTR	0.07	0.05	0.04	0.01	0.24	0.05	0.83
	N3FA	0.06	0.03	0.04	0.01			
		a	ab	b				
Tricosanoic^c^	CTR	0.04	0.02	0.02	0.01	0.81	0.01	0.86
	N3FA	0.04	0.02	0.02	0.01			
		a	b	b				
**Monounsaturated fatty acids, g/100 g**								
cis-10-heptadecenoic	CTR	0.33	0.44	0.51	0.06	0.72	0.03	0.84
	N3FA	0.38	0.47	0.51	0.07			
		a	b	b				
Vaccenic^c^	CTR	0.91	0.85	0.65	0.10	0.73	0.04	0.90
	N3FA	0.88	0.78	0.65	0.11			
		a	a	b				
Oleic	CTR	9.09	11.89	12.33	0.70	0.97	<0.01	0.13
	N3FA	8.90	13.09	11.42	0.70			
		a	b	b				
Nervoic^c^	CTR	0.10	0.04	0.17	0.04	0.59	0.07	0.87
	N3FA	0.12	0.07	0.16	0.04			
		ab	a	b				
**Omega-6 polyunsaturated fatty acids, g/100 g**								
Linolelaidic	CTR	0.17	0.11	0.07	0.03	0.40	0.01	0.50
	N3FA	0.23	0.09	0.11	0.04			
		a	b	b				
Rumenic^c^	CTR	0.13	0.17	0.17	0.01	0.94	<0.01	0.16
	N3FA	0.13	0.18	0.15	0.01			
		a	b	b				
CLA isomers^c^	CTR	1.20	0.89	0.64	0.13	0.52	0.02	0.56
	N3FA	1.16	0.95	0.88	0.15			
		a	ab	b				
GLA^c^	CTR	0.91	0.70	0.54	0.09	0.15	0.09	0.19
	N3FA	0.90	0.80	0.87	0.10			
		a	ab	b				
DGLA^c^	CTR	2.11	1.49	1.24	0.10	0.57	<0.01	0.76
	N3FA	2.11	1.35	1.23	0.12			
		a	b	b				
Docosadienoic^c^	CTR	0.66	0.38	0.30	0.06	0.63	<0.01	0.25
	N3FA	0.74	0.28	0.21	0.07			
		a	b	b				
**Sums and ratios** ^**6**^								
SFAs^c^	CTR	43.5	40.8	38.3	1.13	0.12	<0.01	0.43
	N3FA	43.1	39.7	34.9	1.30			
		a	b	c				
PUFAs^c^	CTR	40.9	41.2	43.1	1.56	0.57	0.01	0.17
	N3FA	41.3	39.8	47.0	1.72			
		a	a	b				
UFAs^c^	CTR	56.5	59.2	61.7	1.13	0.12	<0.01	0.43
	N3FA	56.9	60.3	65.1	1.30			
		a	b	c				
SFA/UFA^c^	CTR	1.07	1.00	0.92	0.06	0.30	<0.01	0.28
	N3FA	1.04	1.00	0.74	0.07			
		a	a	b				
n6/n3	CTR	6.76	7.70	9.25	0.77	0.17	0.04	0.40
	N3FA	6.40	6.17	7.17	0.82			
		a	a	b				

1 *CLA: conjugated linoleic acid, GLA: gamma-linoleic acid, DGLA: dihomo gamma-linoleic acid, SFAs: saturated fatty acids, UFAs: unsaturated fatty acids, PUFAs: polyunsaturated fatty acids*;

c *parameter was covariate on−3 DFC value*.

2 *Treatment (CTR is cows receiving physiological solution and n3FA is cows receiving emulsified fish oil as intrajugular infusion)*.

3 *Time: a, b, c indicates differences between time points (P < 0.05). Time points having the same letters are equal*.

4 *Treatment X time interaction effect*.

5 *Standard error: largest standard error for the fixed effects*.

6 *g/100 g for total SFAs, PUFAs and UFAs*.

#### Monounsaturated Fatty Acids

While the proportion of *cis*-10-pentadecanoic acid (C15:1-*cis*) increased throughout the experimental period (Figure 3C), myristoleic (C14:1), *cis*-10-heptadecanoic (C17:1-*cis*), and oleic (C18:1-*cis*) acids increased at 3 DFC and remained constant through lactation (Figure 3B, Table 4). Palmitoleic acid (C16:1-*cis*) increased at 10 DFC compared to−7 DFC (Figure 3D). While the proportion of nervonic acid (C24:1) increased, vaccenic acid (C18:1-trans11) decreased at 10 DFC (Table 4). The proportion of myristoleic acid was higher, and palmitoleic acid tended to be higher in n3FA than the CTR group (*P* < 0.01 and *P* = 0.07; Figures 3B,D). The proportion of myristoleic and *cis*-10-pentadecanoic acids were affected by a TRT × time interaction (*P* = 0.05 and *P* < 0.01, respectively; Figures 3B,C). Compared to CTR, the n3FA group had a higher proportion of myristoleic acid at 3 and 10 DFC and a lower proportion of *cis*-10-pentadecanoic acid at 10 DFC (*P* < 0.01). No differences over time or between groups appeared for the concentration of C18:1 trans and cis isomers (other than vaccenic) and cis-11-eicosenoic acid (C20:1) (data not shown).

#### Omega-6 Polyunsaturated Fatty Acids

The proportion of rumenic acid (C18:2n-6-cis9, trans11) increased at 3 DFC and remained constant through lactation. But linolelaidic (C18:2n6-trans), CLA isomers (other than rumenic acid), gamma-linolenic acid (GLA – C18:3n-6), *cis*-11,14-eicosadienoic (C20:2n-6-cis), dihomo-gamma-linolenic acid (DGLA – C20:3n-6-*cis*), and docosadienoic (C22:2n-6-*cis*) acids decreased at 3 DFC and remained constant through lactation (Table 4, Figure 3F). The proportion of linoleic acid (C18:2n-6-*cis*) increased at 10 DFC compared to 3 DFC and had a tendency toward a TRT x time interaction (*P* = 0.09; Figure 3E). The proportion of cis-11,14-eicosadienoic tended to be lower in n3FA than in the CTR group and was affected by a TRT x time interaction (*P* = 0.09 and *P* = 0.05; Figure 3F). Compared to CTR, the n3FA group had a higher proportion of *cis*-11,14-eicosadienoic acid at 3 DFC (*P* < 0.01). No differences over time or between groups appeared for the concentration of arachidonic acid (ARA – C20:4n6-*cis*) (data not shown).

#### Omega-3 Polyunsaturated Fatty Acids

The proportion of EPA increased at 3 DFC and remained constant at 10 DFC (Figure 3G). The proportion of EPA tended to be higher (*P* = 0.09; Figure 3G), and DHA's were higher in n3FA than in the CTR group (*P* < 0.01; Figure 3H). The proportion of DHA was affected by a TRT x time interaction (*P* = 0.03; Figure 3H). Compared to CTR, the n3FA group had a higher proportion of DHA at 3 and 10 DFC (*P* < 0.05 and *P* < 0.01). No differences over time or between groups appeared for the concentration of alpha-linolenic acid (ALA – C18:3n-3-*cis*) and *cis*-11,14,17-eicosatrienoic acid (C20:3n-3-*cis*) (data not shown).

#### Sums and Ratios

The total amount of MUFAs increased at 3 DFC and remained constant at 10 DFC (Figure 3I). The total amount of PUFAs and the n6/n3 ratio increased at 10 DFC compared to 3 DFC (Table 4). The total amount of SFAs decreased, unsaturated FAs increased throughout the experimental period, and the SFA/UFA ratio decreased at 10 DFC (Table 4). The total amount of MUFAs was affected by a TRT x time interaction (*P* = 0.04; Figure 3I). Compared to CTR, the n3FA group tended toward a higher proportion of MUFAs at 3 DFC (*P* < 0.1).

### Plasma Metabolic Profile

#### Packed Cell Volume, Energy, Protein, and Mineral Metabolism Biomarkers

**Figure 4:**
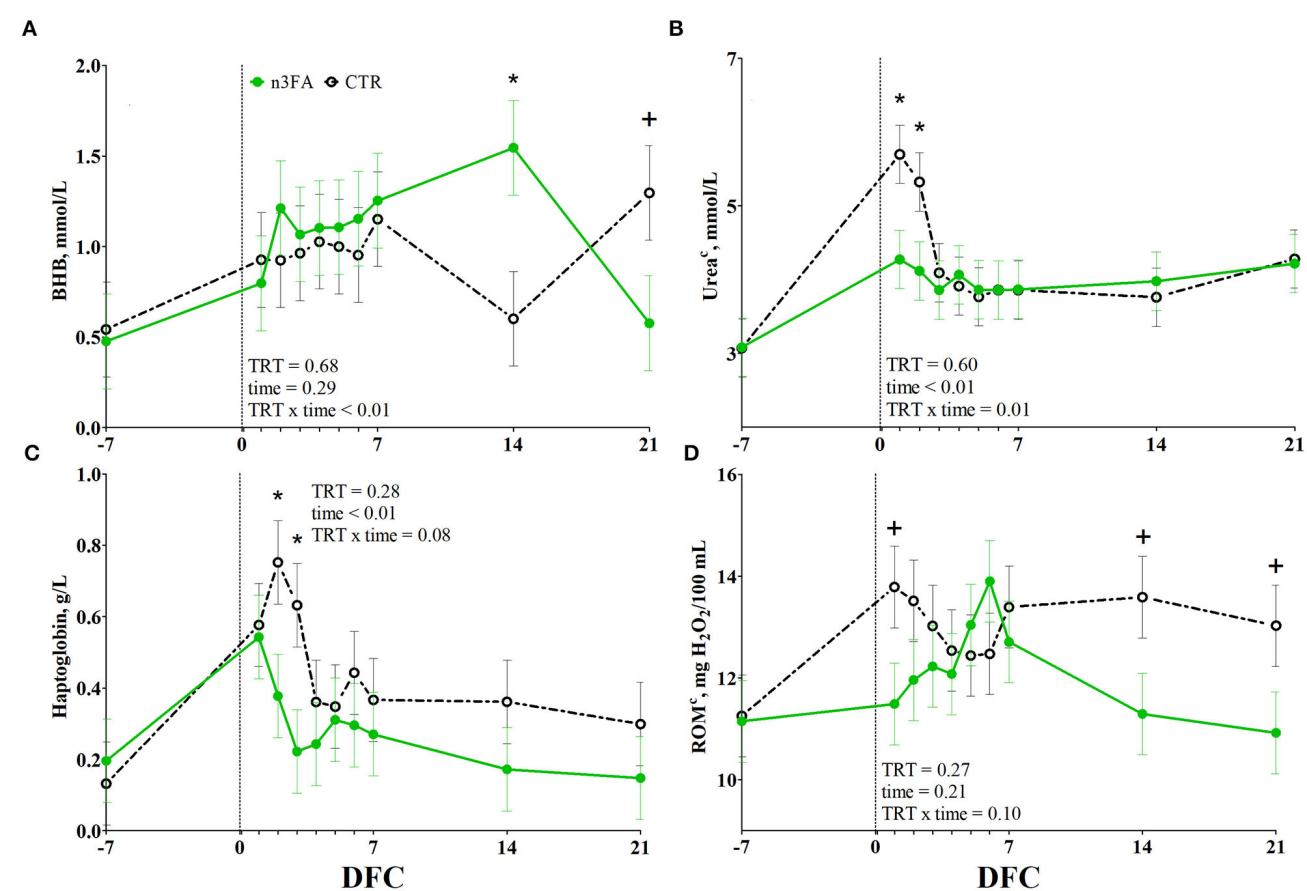
Time course of plasma concentrations of beta-hydroxybutyrate (**BHB**; **A**), urea (**B**), haptoglobin **(C)**, and total reactive oxygen species (**ROMt**; **D**) in dairy cows infused with 150 ml of sterile saline (CTR; black dotted line) or 150 ml of a 10% solution containing purified fish oil rich in long-chained omega-3 fatty acids (n3FA; green solid line) at 12, 24, and 48 h after calving. TRT is treatment effect; TRT x time is treatment x time interaction effect (* is *P* < 0.05; + is *P* < 0.10), c parameter was covariate on−3 DFC value.

Among the energy metabolism biomarkers, BHB was affected by a TRT x time interaction (*P* < 0.01; Figure 4A). Compared to CTR, n3FA group had a higher BHB concentration at 14 DFC and a tendency toward a lower BHB concentration at 21 DFC (*P* < 0.05 and *P* < 0.1). No difference between the groups appeared for packed cell volume, glucose, and NEFA concentrations (Supplementary Figures 1A–C).

Among the protein metabolism biomarkers, urea was affected by a TRT x time interaction (*P* = 0.01; Figure 4B). Compared to CTR, n3FA group had lower plasma urea concentration at 1 and 2 DFC (*P* < 0.05). No effect was detected for creatinine or Zn concentration (Supplementary Figures 1D,E).

#### Liver Function and Inflammation Biomarkers

Among the inflammation biomarkers, haptoglobin tended toward a TRT x time interaction (*P* = 0.08; Figure 4C). Compared to CTR, n3FA group had lower concentration of haptoglobin at 2 and 3 DFC (*P* < 0.05). No difference between the groups appeared for liver enzymes and the remaining inflammation biomarkers (Supplementary Figures 1F–H, 2A–G).

#### Oxidative Status Biomarkers

Among the oxidant species, ROMt tended toward a TRT × time interaction (*P* = 0.1; Figure 4D). Compared to CTR, the n3FA group tended to lower ROMt concentration at 1, 14, and 21 DFC (*P* < 0.1). No difference between groups appeared for SHp concentration (Supplementary Figure 2H), β-carotene, and tocopherol (data not shown).

### Milk Quality and Efficiency Measurements

Among the milk composition measurements, lactose and urea-N were lower in the n3FA than in the CTR group (*P* =0.04 and *P* = 0.01; Table 5). Among milk rheological parameters, rCT and a30 were affected by a TRT x time interaction (*P* = 0.05 and *P* = 0.03; Table 5). Compared to CTR, n3FA group had a lower rCT and a higher a30 at 3 DFC (*P* < 0.01). No difference between groups appeared for the other milk quality and efficiency measurements (Table 5).

**Table 5:** Milk composition, somatic cells count and rheological measurements, and feed efficiency measurement in dairy cows infused with 150 ml of sterile saline or 150 ml of a 10% solution containing purified fish oil rich in long-chained omega-3 fatty acids at 12, 24, and 48 h after calving.

**Item, Unit^**a**^**	**TRT^**b**^**	**Days from calving**						**SEM^**d**^**	***P*** **-Value**		
		**3**	**7**	**10**	**14**	**17**	**21**		**TRT^**b**^**	**time**	**TRT X time^**c**^**
**Composition**											
Butterfat,	CTR	5.01	4.87	4.44	4.02	4.01	3.87	0.25	0.53	<0.01	0.51
g/100 g	n3FA	5.00	4.91	4.68	4.55	4.17	3.73				
Total protein,	CTR	4.42	3.62	3.36	3.10	3.13	2.98	0.11	0.73	<0.01	0.53
g/100 g	n3FA	4.20	3.74	3.44	3.28	3.09	3.06				
True protein,	CTR	4.23	3.45	3.20	2.94	2.98	2.82	0.12	0.44	<0.01	0.45
g/100 g	n3FA	4.05	3.63	3.32	3.17	2.97	2.93				
Lactose,	CTR	4.48	4.77	4.89	4.98	5.01	5.02	0.07	0.04	<0.01	0.87
g/100 g	n3FA	4.40	4.60	4.75	4.77	4.90	4.90				
Casein,	CTR	3.25	2.69	2.49	2.30	2.33	2.21	0.08	0.98	<0.01	0.71
g/100 g	n3FA	3.10	2.72	2.52	2.41	2.29	2.24				
Titratable acidity,	CTR	4.95	4.22	3.92	3.73	3.60	3.65	0.23	0.94	<0.01	0.93
°SH/50 mL	n3FA	5.16	4.29	3.87	3.69	3.65	3.51				
Urea-N,	CTR	28.8	25.1	23.5	24.2	22.8	24.3	1.90	0.01	<0.01	0.79
mg/dL	n3FA	25.0	18.4	19.3	19.1	20.4	21.1				
SCC,	CTR	12.9	12.1	11.4	11.7	11.8	12.1	0.66	0.38	<0.01	0.91
LS	n3FA	13.6	12.7	12.1	12.0	12.4	11.9				
**Outputs, ratios, and efficiency measurements**											
Fat output,	CTR	1311.6	1529.5	1478.7	1576.2	1591.2	1600.3	175.7	0.90	0.04	0.34
kg	n3FA	1424.9	1533.1	1628.3	1533.6	1565.1	1470.9				
Total protein output,	CTR	1154.8	1149.4	1138.6	1225.2	1238.6	1239.6	129.2	0.96	0.18	0.21
kg	n3FA	1202.7	1172.5	1204.4	1175.9	1176.4	1228.2				
FPR,	CTR	1.15	1.34	1.32	1.29	1.27	1.29	0.07	0.58	<0.01	0.54
-	n3FA	1.12	1.24	1.30	1.33	1.29	1.16				
ECM,	CTR	32.9	37.0	37.1	40.8	41.3	41.9	12.62	0.94	<0.01	0.20
kg	n3FA	35.4	37.4	40.1	38.7	40.1	40.2				
FE,	CTR	2.61	2.36	2.21	2.28	2.19	2.12	0.18	0.79	0.01	0.89
-	n3FA	2.56	2.41	2.45	2.30	2.25	2.14				
**Rheological measurements**											
rCT,	CTR	22.4	12.1	13.1	14.1	14.5	15.7	2.00	0.05	0.02	<0.01
min	n3FA	9.3	9.3	12.5	12.8	13.8	16.4				
		**									
a30,	CTR	19.4	34.2	33.8	31.7	30.3	27.5	4.18	0.03	0.08	0.02
mm	n3FA	45.1	43.1	37.3	33.5	30.0	26.4				
		**									

a FPR: fat to protein ratio = Butterfat (g/100 g)/Total protein (g/100 g); ECM: energy corrected milk = (milk yield (kg/day) ^*^ (0.383 ^*^ Butterfat (g/100 g) + 0.242 ^*^ Total protein (g/100 g) + 0.1571 ^*^ Lactose (g/100g) + 0.207) / 3.140); FE: feed efficiency = ECM (kg/day)/Dry matter intake (kg/d); rCT: Rennet clotting time; a30: curd firmness; SCC: Somatic cells count; LS: linear score= Log_2_[(SCC/12.500) – 1].

b *Treatment (CTR is cows receiving physiological solution and n3FA is cows receiving emulsified fish oil as intrajugular infusion)*.

c Treatment X time interaction (^**^ is P < 0.01 for differences among means within a column. These symbols are only presented when the interaction effect is significant).

d *Standard error: largest standard error for the fixed effects*.

## Discussion

At the plasma total lipids profile level, n3FA cows had higher plasma proportions of EPA and especially of DHA at 3 DFC compared to CTR cows, and they maintained them until 10 DFC (at 24 h and 8 days after the last administration). Previous studies reported no trace of EPA and a low and stable proportion of DHA in the plasma total lipids FAs profile of dairy cows at their calving transition (11), and the outcomes obtained in this study are thus probative that fish oil infusion effectively elevates the availability of these long-chain n-3 PUFAs in plasma of postpartum cows. Such an elevation of EPA and DHA abundance detected in plasma aligns with the improved rheological parameters (i.e., reduced rCT and increased a30) of the milk from n3FA cows at 3 DFC. Changes in the FAs profile of plasma are reflected by those of the milk fat (16), and higher inclusion of long-chained PUFAs in the milk fat positively affects the cheesemaking features, improving the cheese yield (48). This also suggests that long-chained n3-PUFAs administered through venal infusion are partially diverted to the mammary gland, which could reduce their plasma availability over time.

The occurrence of a systemic inflammatory state is a recurring condition in dairy cows at the onset of lactation (3, 49). The lowered ROMt concentration found in the plasma of n3FA cows at 1 DFC and the lowered haptoglobin concentration at 2 and 3 DFC suggest that the fish oil infusion is effective at mitigating their systemic inflammatory state as compared to CTR cows. In fact, ROMt elevation in plasma of early lactating cows could be driven by leukocytes activation during inflammation (50), while haptoglobin is the main acute-phase protein in cows, and it serves as a systemic inflammation marker by reflecting an acute-phase response occurring at the liver level (51, 52).

The mitigated inflammatory conditions of n3FA cows could likely be driven by a greater EPA and DHA inclusion in the PLMs of their leukocytes as compared to CTR cows. In fact, the enrichment of leukocytes PLMs with EPA and DHA is known for playing an anti-inflammatory action through inhibiting the release of proinflammatory mediators (i.e., prostaglandins) and altering the formation of lipid rafts involved in NF-kB activation (19). Despite the plasma total lipids FAs profile assessed in the present study only reflects the changes in the neutral lipids and NEFA fractions, it could be hypothesized that EPA and DHA elevation detected at the plasma total lipids level could have come with an even greater elevation of these n3PUFAs in the leukocytes PLMs. In fact, plasma FAs are differentially spared among the body compartments depending on the length of their carbon chains (11, 53). Long-chained PUFAs are preferentially included in the PLMs (54, 55). Among these, EPA and DHA are known for their high inclusion rate in the PLM of leukocytes at the expense of ARA (an n6-FA representing the main PUFA composing leukocytes PLMs in normal conditions) (19, 32).

Results obtained in this study contradict those obtained when administering n3 PUFAs through diet and that report no effect on any acute-phase protein biomarker (21, 56). Thus, greater effectiveness of the venal infusion in favoring the inclusion of n3 PUFA in the leukocytes PLMs could be hypothesized, suggesting that it is a potential strategy to cope with limitations hindering lipid nutrition in dairy ruminants. The short time frame occurred between calving and the administration of the first lipid infusion could also account for the effectiveness of n3 PUFA in mitigating plasma haptoglobin elevation in this study, as acute inflammation driven by calving is reflected by a massive haptoglobin peak in healthy dairy cows during the first few days after calving (57). Despite that, haptoglobin was the only inflammatory marker affected by the infusion in the present study, and no effect was found on the other acute-phase proteins (i.e., ceruloplasmin and negative acute-phase proteins). We can thus speculate that the amount of n3 PUFA provided by the 3 intravenous infusions is not sufficient to fully overcome the inflammatory condition faced by dairy cows at the onset of lactation.

The effectiveness of the fish oil infusion in mitigating the systemic inflammatory state of n3FA cows could account for the higher DMI they had during the first week of lactation as compared to CTR cows because cytokines tuning the inflammatory processes reduce feed intake through exerting an anorexic power on the hypothalamus in periparturient dairy cows (58). We can speculate that the greater DMI of the n3FA cows at the onset of lactation could have modified the relative abundances of dietary- and lipomobilization-related FAs in their plasma profile as compared to those of CTR cows (i.e., increased abundance of MUFAs at 3 DFC and increased abundance of *cis*-11,14-eicosadienoic acid counterbalanced by a reduced abundance of myristic acid and *cis*-10-pentadecanoic acid at 10 DFC). Yet a detailed discussion of these differences from a metabolic standpoint could be hard to assess because little is known about the metabolic pathways regulating minor FA abundance in the plasma of dairy cows (11). Besides inducing changes in the FA profile of n3FAcows, greater feed intake of these animals could account for their greater milk yield recording and lower plasma urea at 1 and 2 DFC, suggesting they have greater energy availability to sustain lactation requirements and a lower amount of endogenous amino acids deserved to gluconeogenetic processes at the beginning of lactation (59, 60) as compared to CTR cows. Although only numerical trends were detected afterward, greater milk yield was maintained by the n3FA cows during the whole experimental period, suggesting that the lower lactose and urea nitrogen content of their milk is driven by a dilution effect. While the reason behind the BHB elevations detected in n3FA cows at 14 DFC and CTR cows at 21 DFC remains unclear, but it seems to be independent of the lipid infusion and likely consequential to the adaptative response of the animals against other minor stressors that occurred along with the lactation.

## Conclusions

The elevation of long-chained n3 PUFAs observed at the plasma total lipid profile level until 10 DFC demonstrated lipid infusion to have a long-lasting effect on the FAs' availability. This could account for an anti-inflammatory action exerted on leukocytes and resulting in mitigating oxidant species release and in the lowered inflammatory status observed in n3FA cows after calving. These alterations in inflammation-related plasma analytes accompanied a higher feed intake and milk yield, confirming that the performances of dairy cows at the onset of lactation largely depend on the severity of the inflammatory conditions they experienced in this phase (61).

Despite these promising outcomes, haptoglobin was the only inflammatory marker affected by the infusion, and no effect was found on the other acute-phase proteins, suggesting that the transient effects of n3PUFAs infusions on plasma analytes are not long lasted and are not very impactful to improve overall inflammatory status within the period assessed by the study. Furthermore, the small sample size suggests the present research to be considered a pilot study, and further experiments testing the effect of a similar lipid infusion performed for a longer time frame and on a wider experimental sample are required to confirm the effectiveness of n3PUFAs infusion at modulating the inflammatory conditions experienced by peripartum dairy cows.

## Data Availability Statement

The raw data supporting the conclusions of this article will be made available by the authors, without undue reservation.

## Ethics Statement

The animal study was reviewed and approved by Authorization of Italian Health Ministry N 1047/2015-PR.

## Author Contributions

FP-C, AM, and ET: conceptualization, methodology, writing—review and editing, and supervision. MM: formal analysis, investigation, and writing—original draft preparation. ET: resources and funding acquisition. MM and FP-C: data curation. FP-C and ET: project administration. All authors have read and agreed to the published version of the manuscript.

## Funding

Research supported by the Università Cattolica del Sacro Cuore Line D.1 – 2011 on Studio sulla utilità della somministrazione di acidi grassi omega-3 nelle bovine da latte.

## Conflict of Interest

The authors declare that the research was conducted in the absence of any commercial or financial relationships that could be construed as a potential conflict of interest.

## Publisher's Note

All claims expressed in this article are solely those of the authors and do not necessarily represent those of their affiliated organizations, or those of the publisher, the editors and the reviewers. Any product that may be evaluated in this article, or claim that may be made by its manufacturer, is not guaranteed or endorsed by the publisher.
