# Supplementary material for: Effects of an Intravenous Infusion of Emulsified Fish Oil Rich in Long-Chained Omega-3 Fatty Acids on Plasma Total Fatty Acids Profile, Metabolic Conditions, and Performances of Postpartum Dairy Cows During the Early Lactation
Source: Front Vet Sci. 2022 May 16;9:870901. doi: 10.3389/fvets.2022.870901 (PMC9149583; doi:10.3389/fvets.2022.870901)
Supplement: Supplementary file 1 [file Data_Sheet_1.pdf]

Supplementary Material

1 Supplementary Tables

Table S1. Intra- and inter-assay coefficient of variations, limit of quantification (LOQ), codes of commercial kits used, references for their validation in the bovine plasma, calibrators and quality controls used for plasma parameters included in the study.

Parameter	Inter	Intra	LOQ	Kit	Reference	Calibrator	Quality control
Glucose	0.75	1.33	0.10	0018250840[1]	-	Homemade bovine standard	Homemade bovine standard; SeraChem Control Level 1, 0018162412[1]; Bov Asy Control 2, AN1026[3]
Cholesterol	1.38	1.56	0.10	0018250540[1]	-		
Urea	1.23	1.14	0.01	0018255440[1]	-		
Total proteins	2.87	1.07	10.00	00182514401	-		
Albumins	3.41	1.20	16.00	00182500401	-		
Creatinine	3.09	1.09	18.00	00182555401	-		
Zinc	3.83	1.76	0.500	439-14906[2]	-	Bov Asy Control 2[4]	
Ceruloplasmin	3.57	1.39	0.100	-	(Sunderman and	Human plasma	

					Nomoto, 1970)	ceruloplasmin, A50143H[5]	
AST / GOT	3.55	1.78	1.00	0018257540[1]	-	No calibration is required	
GGT	4.42	2.27	2.00	0018257640[1]	-		
Paraoxonase	4.60	1.38	1.20	-	(Ferré et al., 2002)		
Haptoglobin	3.50	3.64	0.01	-	(Skinner et al., 1991)		
Total Bilirubin	2.46	3.11	2.00	0018254640[1]	-	Bov Asy Control 2[4]	
Alkaline phosphatase	3.52	0.77	2.00	0018259640[1]	-	ReferrIL E, 0018256300[1]	
NEFA	5.00	2.27	0.010	NEFA-HR(2) R1 Set, 434-91795[2]	-	NEFA standard, 270-77000[2]	
BHB	3.42	0.82	0.100	RB1007[3]	-	BHB standard included in the kit	
ROMt	2.93	1.40	3.20	MC003[4]	-	Calibrator d-ROMs, MC.030[6]	Homemade bovine standard

	SeraChem Control Level 1, 0018162412[1]; Control Serum, MC03[4]; Calibrator d-ROMs, MC030[4]

[1]Instrumentation Laboratory Werfen, Milano, Italy;
[2]Wako Chemicals GmbH, Neuss, Germany;
[3]Randox Laboratories Ltd., Crumlin, County Antrim, UK;
[4]Diacron International S.r.l., Grosseto, Italy;
[5]Meridian Life Science, Memphis, USA;
[6]Carlo Erba, Rodano, Milano, Italy.

2 Supplementary Figures

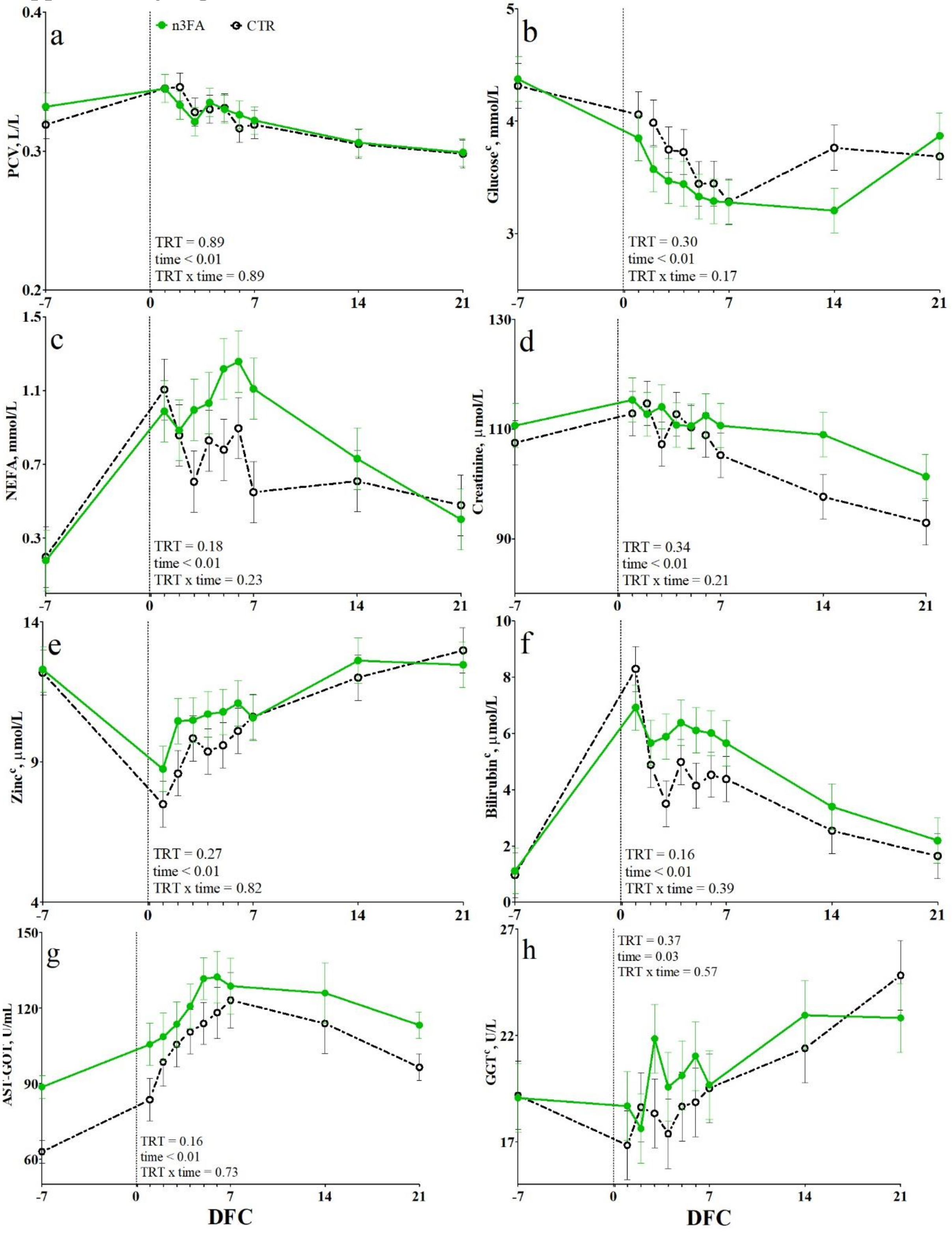

Supplementary Figure 1. Time course of packed cell volume (PCV; a) and plasma concentrations of glucose (b), nonesterified fatty acids (NEFA; c), creatinine (d), zinc (e), bilirubin (f), aspartate aminotransferase – glutamate oxaloacetate transaminase (AST-GOT; g) and γ-glutamyl transferase (GGT; h) in dairy cows infused with 150 mL of sterile saline (CTR; black dotted line) or 150 mL of a 10% solution containing purified fish oil rich in long chained omega 3 fatty acids (n3FA; green solid line) at 12, 24 and 48 h after calving. TRT is treatment effect; TRT x time is treatment x time interaction effect, c parameter was covariate on -3 DFC value.

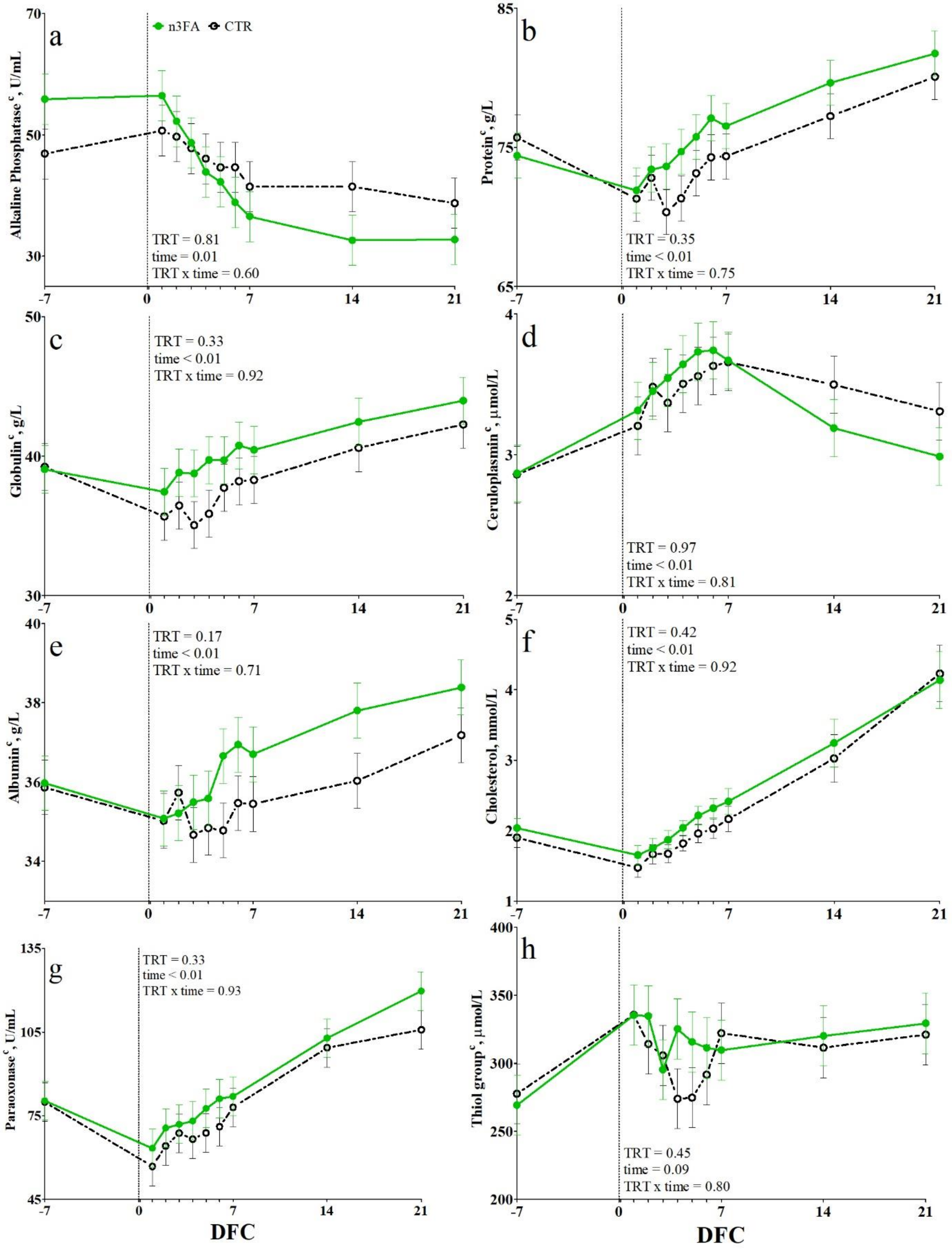
a
n3FA
CTR
Alkaline Phosphatase c, U/mL
TRT = 0.81
time = 0.01
TRT x time = 0.60
b
Protein c, g/L
TRT = 0.35
time < 0.01
TRT x time = 0.75
c
Globulin c, g/L
TRT = 0.33
time < 0.01
TRT x time = 0.92
d
Ceruloplasmin c, µmol/L
TRT = 0.97
time < 0.01
TRT x time = 0.81
e
Albumin c, g/L
TRT = 0.17
time < 0.01
TRT x time = 0.71
f
Cholesterol, mmol/L
TRT = 0.42
time < 0.01
TRT x time = 0.92
g
Paraoxonase c, U/mL
TRT = 0.33
time < 0.01
TRT x time = 0.93
h
Thiol group c, µmol/L
TRT = 0.45
time = 0.09
TRT x time = 0.80
DFC
DFC

Supplementary Figure 2. Time course of plasma concentrations of alkaline phosphatase (a), protein (b), globulin (c), ceruloplasmin (d), albumin (e), cholesterol (f), paraoxonase (g) and thiol groups (h) in dairy cows infused with 150 mL of sterile saline (CTR; black dotted line) or 150 mL of a 10% solution containing purified fish oil rich in long chained omega 3 fatty acids (n3FA; green solid line) at 12, 24 and 48 h after calving. TRT is treatment effect; TRT x time is treatment x time interaction effect, c parameter was covariate on -3 DFC value.
